# Effect of Various Types of Superplasticisers on Consistency, Viscosity, Structure and Long-Term Strength of Geopolymer Products

**DOI:** 10.3390/ma14247614

**Published:** 2021-12-10

**Authors:** Łukasz Anaszewicz

**Affiliations:** Faculty of Civil Engineering and Geodesy, Military University of Technology in Warsaw, 00-908 Warsaw, Poland; lukasz.anaszewicz@wat.edu.pl

**Keywords:** plasticisers, geopolymer, consistency, viscosity, strength, microstructure

## Abstract

This article presents the results of research on the effect of plasticisers made based on four different compounds—melamine (M), naphthalene (NF), acrylic polymers (AP) and polycarboxylic ethers (PC)—added to the tested mixes in the amount of 2% of the fly ash (FA). The influence of superplasticisers (SPs) on the consistency of the fresh concrete was investigated using a flow table and a penetrometer, and the air voids content was determined by means of a porosimeter. Additionally, the influence of plasticisers on the viscosity of the paste was investigated using a rheometer. Hardened mortar that matured under two different conditions was also tested at elevated and room temperatures. The tested properties were 7-, 28- and 90-days compressive strength and internal microstructure viewed under a microscope. NF had the greatest viscosity-reducing effect while it increased the air void volume in the mix at the same time. The highest early and late strengths were obtained after curing in elevated temperature samples with an acrylic-polymer-based superplasticiser. However, the increased curing temperature of the samples only influenced the early strength results. Its effect was not visible after 90 days. The AP addition also had a significant impact on improving the consistency of the mixture. The addition of plasticisers did not affect the microstructure of the specimens.

## 1. Introduction

In the era of increasing demand for cement, we have reached the point where there are shortages of this product on the market. This situation increases the need for the quick phasing in of products that could replace cement-based concrete. Dusty industrial waste materials are an increasingly well-researched alternative material in this application. The energy economy, so far based to a large degree on coal, has provided us with fly ash (FA) deposits, which require disposal and at the same time are a promising alternative to cement. This is clearly demonstrated by the results of research on geopolymer mortars [1,2,3], concretes [4,5,6,7] and even models of structural elements [8,9,10].

However, even though geopolymeric materials intended for use in construction have been studied for a long time, there is still a need for well-tested and dedicated workability-improving agents. Until they are developed, we must rely on agents designed for cement concrete available on the market.

One of the first studies in this area is presented in [11], where granular blast furnace slag (GGBFS) geopolymer products were supplemented with 1% plasticiser additive (SP) based on four different compounds: polycarboxylic ethers (PC), melamine (M), naphthalene (NF) and vinyl copolymers (V). The tests demonstrated a slight effect on the 28-day compressive strength of the test specimens (in relation to cement concrete controls), no increase in slump for PC, M and V and a considerable increase in slump in the case of NF specimens. GGBFS appeared in the study reported in [12], in which from 2% to 6% addition of a plasticiser of an unknown basis was used, and the specimens were cured in a room-temperature environment. In this case, the results show an increase in cone slump ranging from 3.7% to 40.7%, depending on the amount of plasticiser, and an increase in compressive strength from 0.4% to 6.1%. Additionally, in [13], the results obtained for geopolymers of variable amounts in the composition of GGBFS and FA were compared. From 1% to 4% of PC- and NF-based plasticisers were added, and the specimens were cured under room-temperature conditions. The study demonstrated that the flow of specimens made with the addition of PC is not only greater, but also lasts longer in relation to the specimens containing N. Additionally, the authors observed that PC improves the 7-day compressive strength of cement base. Another study which presents the results of the geopolymer tests based on GGBFS and on the mix of GGBFS with FA is [14]. The specimens contained 1%, 2% and 3% of PC-based SP. The test results presented by the authors were 7- and 28-day strength data. Both in the case of compressive strength and tensile strength by splitting, higher strengths were obtained for specimens with a higher SP content.

One of the first papers to study fly ash (FA) geopolymers was [15]. Plasticisers based on lignosulfonates (L), melamine (M) and polycarboxylic ethers (PC) were used for the tests. In their conclusions, the authors pointed out that the viscosity of all the specimens was lower than the viscosity of the control, but higher than the viscosity of the specimen containing Portland cement. Moreover, the plasticising properties disappeared in less than 10 min. Additionally, the correlation between the obtained slump and the viscosity data was investigated. According to the authors, using a viscometer for measurements is a more accurate method for studying the influence of admixtures on the behaviour of geopolymers. On the other hand, the slump cone test is only suitable for Portland cement specimens. Studies [16,17] investigated the influence of PC on FA-based geopolymers. Their objective was to obtain self-compacting concrete, and therefore, the amount of plasticiser was between 3% and 7%. The specimens were heated at 70 °C for 24 h and 48 h, respectively. The results show that a higher amount of admixture increased the strength. In addition, the use of an activator with a higher NaOH_sol_ molarity and increasing the amount of the SP additive reduced cracking at the matrix–aggregate interface. The results of tests on specimens containing similar amounts of PC and NF plasticisers, i.e., 1% to 5%, are presented in [18]. Viscosity was tested by means of a viscometer at 5 rpm for a period of 30 min. It was demonstrated that PC plasticiser slightly reduced the viscosity of the paste, in contrast to NF, which increased the viscosity and accelerated the setting time. However, the authors did not investigate the effect of SP on the strength. Rheological properties were also examined in [19]. L- and PC-based plasticisers were used in the tests, added at 1% and 1.5%, respectively. NaOH_sol_ was changed in addition. According to the authors, the molarity of the NaOH_sol_ solution above 4M affects the rheological parameters of the fresh concrete and reduces slump. Better workability results were obtained for L-based plasticisers. The effect of 1% addition of M-, NF- and PC-based plasticisers was examined in [20]. The strength test specimens were heated for 24 h at 60 °C before the test. The slump of specimens in which the two-component activator was used was the greatest, in the range of 39–45% for the PC-based plasticiser; for the NF plasticiser, the increase was of 6–8%. On the other hand, M-based plasticiser gave a 3% reduction in the slump value as compared to the control. At the same time, there was a loss in the strength of the specimens with the SP addition compared to the control. The effect of NF and PC added at 0.5% to 2% was also investigated in [21]. Two types of fly ash were used in this study, i.e., with low and high limestone content (class F and C, respectively). The results show that in the case of low-calcareous ash, the use of a naphthalene plasticiser results in a greater paste plasticisation as compared to PC. In the case of high-calcareous ash, the situation is opposite. Better results were obtained with PC. Additionally, it was observed that in the specimen containing class F ash, the relationship between shear stresses and shear rate was the same for each of the tested superplasticisers. In the case of class C ash, the curve decreased along with the increasing fluidity of the mix. Unfortunately, the authors did not test the specimens for strength. There are publications in the literature that deal with the influence of plasticisers on geopolymers made of less common materials. Study [22] gives the results of specimens from oil palm fibre ash with a 10% addition of an NF-based plasticiser. On the other hand, study [23] gives the results of research regarding one-component geopolymeric concrete. It was prepared by mixing the dry ingredients, including FA, GGBFS and various types of powdered sodium silicate, and then adding water and other liquid additives. PC and NF plasticisers added at 1% were used for the tests, and the strength test specimens were heated prior to testing. The results demonstrate a positive effect of plasticisers on the flow, along with simultaneous reduction in the strength of most of the specimens. Table 1 compiles the plasticisers and the above-mentioned studies. Part of the consistency testing was carried out using the slump cone method, and some was performed using the flow table method.

The review of all these publications makes it possible to narrow down the choice of plasticisers depending on the produced mix. However, none of them are comprehensive enough to define the influence of plasticisers on the consistency and viscosity, as well as the short- and long-term strength of the material depending on the curing conditions. This being so, this article presents the results of laboratory tests on the influence of the four types of plasticisers on the consistency and viscosity of the geopolymer paste and on the 7-, 28- and 90-day strength of the specimens of mortar maturing both at room temperature and heated after moulding for 24 h at the temperature of 70 °C.

## 2. Materials and Production Methodology

### 2.1. Matrix

The specimens contained fly ash (FA) classified as class F as per ASTM C618 or class V ash as per EN 197-1. A mix of sodium silicate aqueous solution and sodium hydroxide solution was used as the activator. The commercial version of water glass R140 with a SiO_2_/Na_2_O molar modulus of 2.9–3.1 was used as the sodium silicate solution (SS_sol_); Na_2_O+SiO_2_ content min. 36% and viscosity (at 20 °C) 0.05 Pa∙s. A sodium hydroxide (NaOH_sol_) solution with a molarity of 12 M was mixed with SS_sol_ at a 1:2.5 ratio.

### 2.2. Admixtures

Four plasticisers, each with different base components, were chosen for use in the tests. Table 2 gives the designations of the specimens along with information on the base component of the plasticiser and the specimen curing conditions. All the tested plasticisers were added at 2% of fly ash by weight.

Melamine and naphthalene-based plasticisers employ an electrostatic effect to fluidise the paste. In simple terms, the process consists of inducing negative charges on the surface of the cement grains, which cause the grains to repel each other. On the other hand, the action of acrylic polymer and polycarboxylic ether-based plasticisers is based on the steric effect. It is associated with long side chains of polymers that make it difficult for the particles to get closer to each other.

### 2.3. Production of the Test Specimens

The specimens were produced using 0–2 mm excavated sand as a filler. For 1 m^3^ of each type of specimen, 160 kg of sand, 53 kg of fly ash, 27 kg of activator, 6 kg of water and 1.06 kg of additives were used to test the consistency, air void content, strength and for a part of the microscopic tests. For the viscosity tests and other microscopic tests, paste of the same proportions was made, except sand was not added to the mix. First, dry ingredients were mixed for 3 min, and then, the prior-mixed liquid ingredients were added to the mix. Then, the whole batch was mixed for another 10 min.

### 2.4. Specimen Curing Conditions

The specimens were divided into two groups. Half of them were cured in a laboratory environment (i.e., at about 20 °C), and the other half were cured at an elevated temperature. The latter were placed in moulds and vibrated and then immediately placed in a lab oven heated up to 70 °C and were kept there for 24 h.

### 2.5. Consistency Testing

The consistency of the fresh concrete was determined in two ways: using a flow table, as per EN 12350-5, and by means of a penetrometer, as per EN 1015-4. The mix was used up within 10 min from the end of mixing. For the flow test, the mix was placed in a truncated-cone-shaped mould, and then, after quick demoulding, the table made 10 strokes and the flow diameter was measured. The penetrometer test consisted of filling the mould with fresh concrete, then lowering the penetrometer’s penetration piston and reading the immersion depth on the gauge. Each mix was subjected to three tests, from which the average value was drawn.

### 2.6. Air Void Content

The air void content was determined upon completion of the mixing. The method prescribed by EN 1015-7 was applied. A 1 L porosimeter was used in the test.

### 2.7. Viscosity

The viscosity test was carried out using a rheometer. The tested paste was composed of activator, water, plasticiser and ash mixed in proportions as for the mortar. Additionally, the test was performed using comparative paste without plasticiser. The ingredients were mixed for 3 min immediately before testing. The test procedure for this test consisted of 10 revolutions of the spindle at speeds from 50 rpm to 200 rpm, and back to 50 rpm at 10 rpm steps. The average result of 10 revolutions for each speed was recorded.

### 2.8. Compressive and Flexural Strength

Mortar strength was tested after 7, 28 and 90 days on 40 mm × 40 mm × 160 mm beam-shaped specimens. The test method of EN 196-1 was used. Four batches that differed with respect to plasticiser were made. Half of the specimens in each batch were cured under laboratory conditions, while the other half were subjected to additional heating immediately after placement in moulds. Each variant was tested for flexural strength on five specimens and for compressive strength on ten specimens.

## 3. Results and Discussion

### 3.1. Consistency Testing

**Figure 1:**
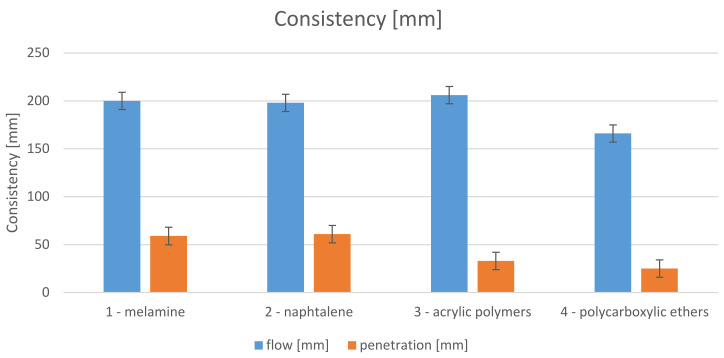
Flow and penetration testing results.

Figure 1 shows that the highest flow of 206 mm was observed on the specimens containing AP acrylic polymers. Still, the difference in flow was not significant when compared to the M specimens—200 mm—and NF specimens—198 mm—containing first-generation plasticisers. The only significantly different result, 166 mm, was obtained for the PC specimen containing a polycarboxylic ether-based plasticiser. The same results for M, NF and PC were obtained in the studies [18,19,21], the last of which indicates that it was related to the use of low-calcareous ash. Opposite results were obtained for high-calcareous ashes.

In the penetration test, the M specimens with 59 mm and NF specimens with 61 mm again achieved a similar rod penetration. On the other hand, the specimens containing AP and PC plasticisers had lower results, i.e., 33 mm and 25 mm in the case of AP and PC, respectively. In the case of M and NF specimens, the penetration rod would easily sink into the fresh concrete, while in the case of the AP and PC specimens, the pressure exerted by the rod pushed the mix sideways and upwards out of the container, and the rod only slightly penetrated the mix.

The above results indicate a lack of a direct correlation between the two methods of fresh mortar consistency testing for different geopolymeric materials. The addition of plasticisers has a desirable effect on the flow of the mix, yet easier penetration was only obtained in the case of M and NF plasticisers.

### 3.2. Air Void Content

**Figure 2:**
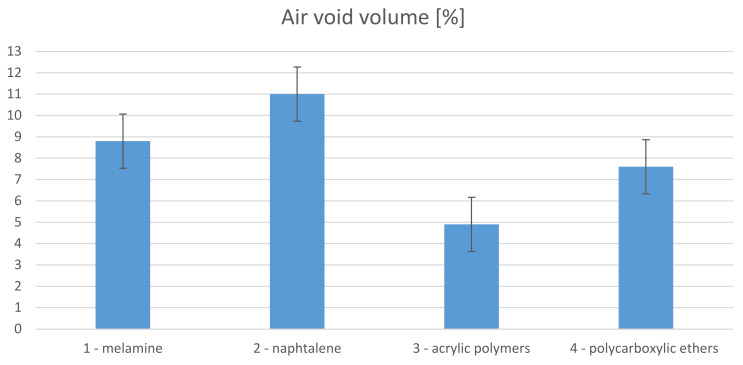
Volume of air voids.

The same conditions were ensured in the preparation of each of the respective mixes. Despite this, Figure 2 shows significant variations in the air void volumes. The AP specimen with 4.9% air void content was the most effectively deaerated specimen. The greatest air void volume, of 11%, was obtained from the NF specimen. No correlation was found with the consistency test results.

### 3.3. Viscosity

**Figure 3:**
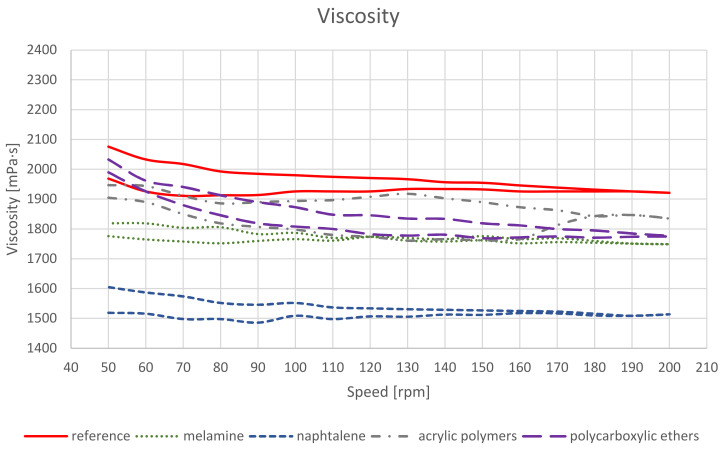
Results of the viscosity tests.

The results given in Figure 3 show that the viscosity of all the mixes containing plasticiser was lower than the control. N had the greatest impact on viscosity reduction, two times the value obtained for M. The differences in comparison to the controls for 200 rpm were 172 and 407 mPa∙s for M and N, respectively. The PC curve overlapped most of the range with the AP curve. The only curve slightly different in course than the reference one was the AP curve. It changed its course while reducing the spindle revolutions. For 130 rpm, the difference was over 100 mPa∙s. This indicates the effect of shear on bonds being formed. Note that this is not evident for high spindle revolution speeds.

A relationship between viscosity and the degree of air entrainment in the mix can be seen: the lower the viscosity, the greater the volume of air voids in the fresh concrete. A relationship between extending the mixing time and increasing the air entrainment of the fresh concrete is known both for cement and asphaltic concretes. As far as geopolymer material is concerned, we can see that the use of additives that strongly lower the viscosity have a significant effect on air entrainment, increasing it considerably. On the other hand, an attempt to reduce air entrainment by shortening the mixing time may lower the strength, which was presented in [4].

### 3.4. Microscopic Testing

The microscopic examination of the structure of the test specimens was carried out on paste specimens prepared in the same way as for viscosity tests and on hardened mortar specimens.

**Figure 4:**
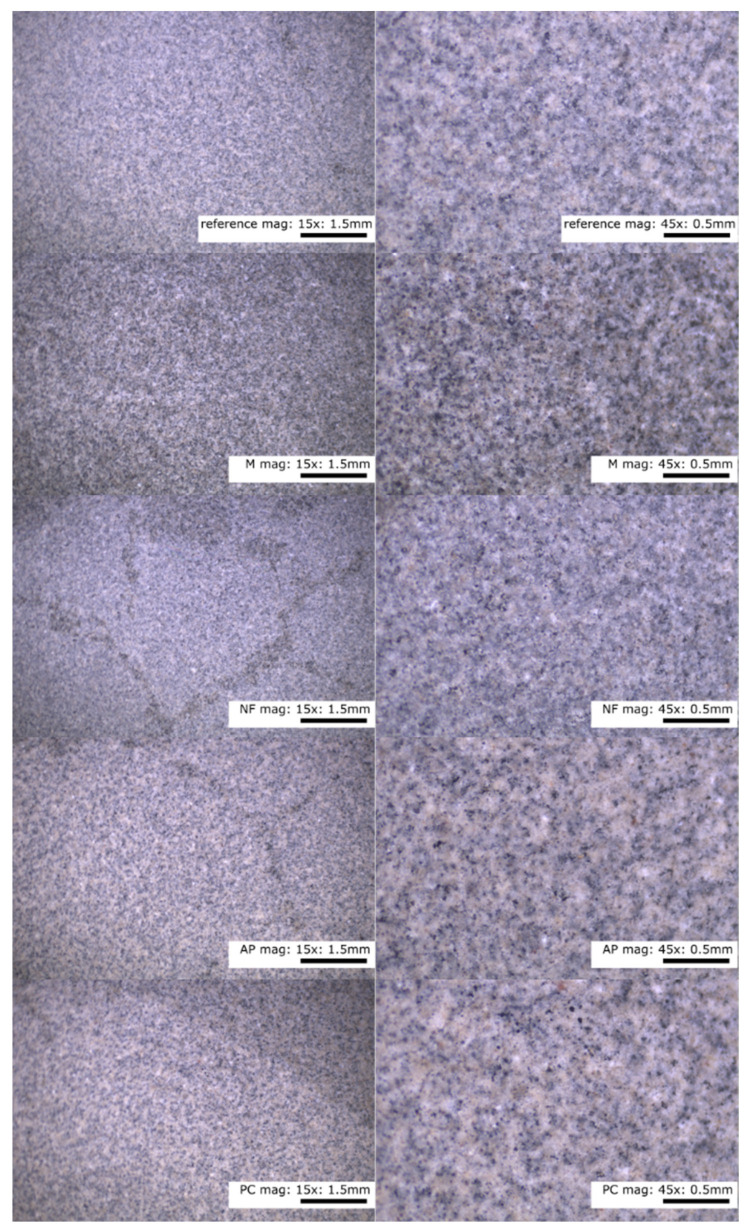
Microscope photographs of the paste at 15× and 45× magnification. Designations: M—melamine, NF—naphthalene, AP—acrylic polymers, PC—polycarboxylic ethers.

The internal structures of the control and of the paste containing the additives were homogeneous throughout. In Figure 4, there are no distinguishable clusters of homogeneous particles or segregation and precipitation of the liquid components of the test mixes.

**Figure 5:**
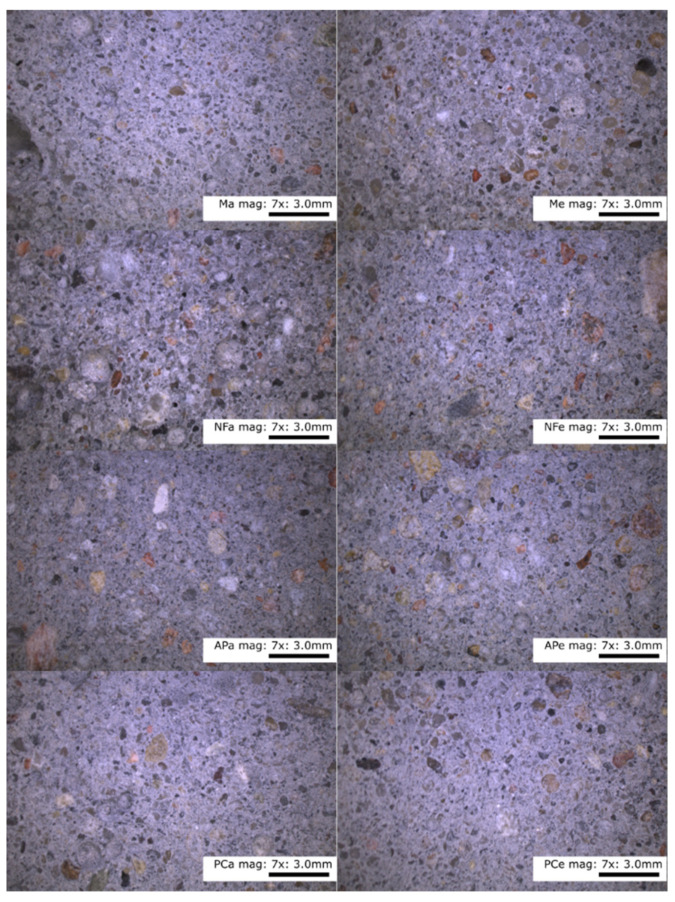
Mortars cured in lab ambient conditions and heated, imaged at 7× magnification. Designations: M—melamine, NF—naphthalene, AP—acrylic polymers, PC—polycarboxylic ethers.

**Figure 6:**
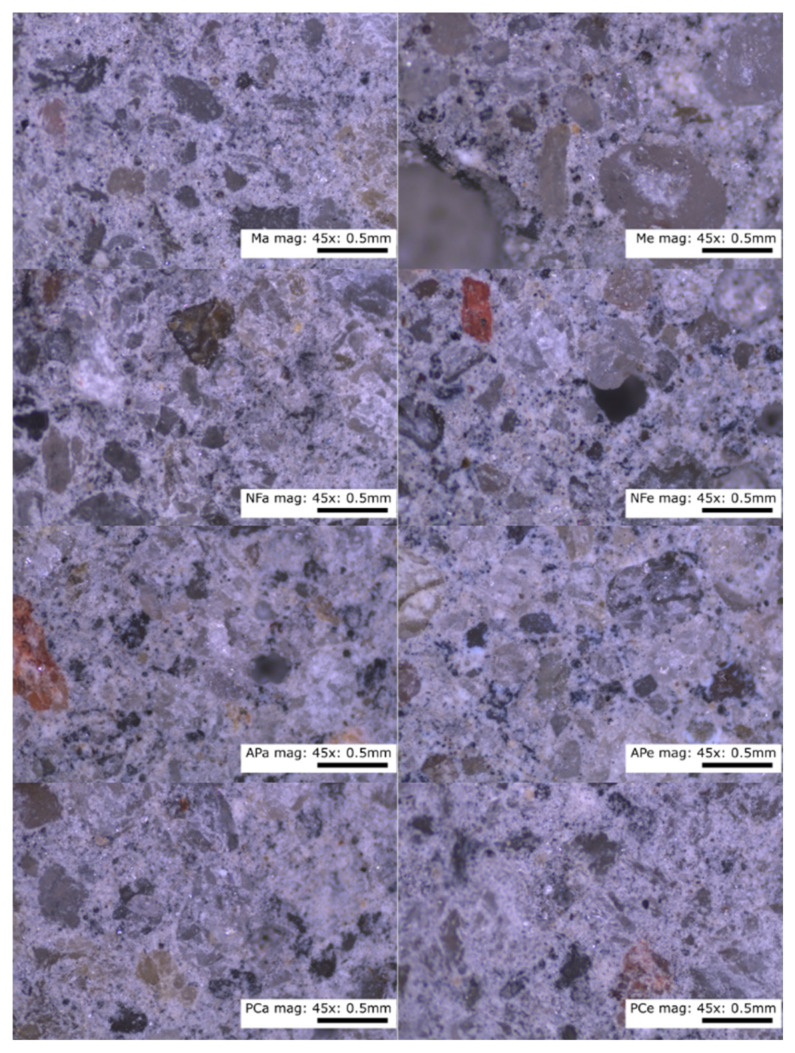
Mortars cured in lab ambient conditions and heated, imaged at 45× magnification. Designations: M—melamine, NF—naphthalene, AP—acrylic polymers, PC—polycarboxylic ethers.

Figure 5 shows mortars at 7× magnification, and even distribution of the aggregate, the same for all additives, can be seen on the images. In the case of N, numerous air voids can be seen. In addition, no shrinkage or interfacial cracking can be seen in Figure 6 at 45× magnification.

Heating the specimens for 24 h at 70 °C had no visible effect on the appearance of the internal structure of the tested specimens. Elevated temperature and faster evaporation did not result in the formation of cracks within the matrix or at the interface between the matrix and the aggregate. None of the specimens showed any signs of precipitation on the surface of the paste specimen, excessive vibration or stratification of the mix components. These results are consistent with observations [24] on the stability of the geopolymer structure at elevated temperatures. The difference in the hues of the pictures is due to the white balance setting.

### 3.5. Compressive and Flexural Strength

**Figure 7:**
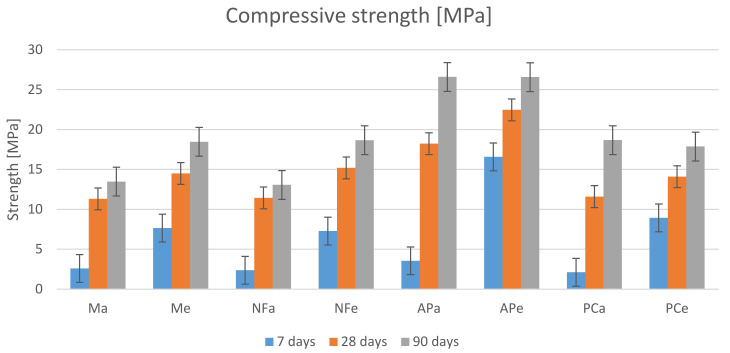
Compressive strength results.

**Figure 8:**
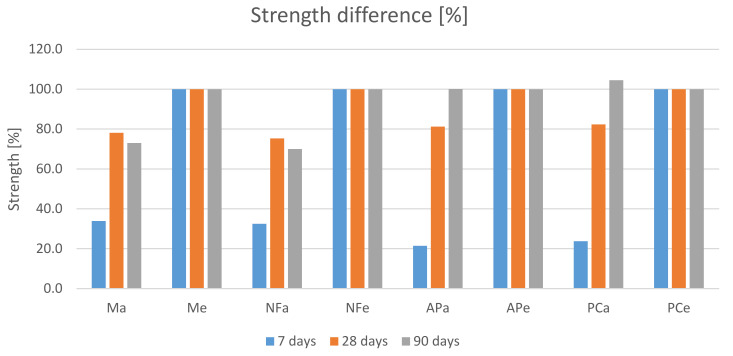
The percentage difference in compressive strength between the heated specimens and the specimens cured under room conditions. To be considered in pairs: Ma-Me, NFa-NFe, APa-Ape and PCa-PCe.

The results shown in Figure 7 indicate a greater increase in 7-day strength for specimens cured at an elevated temperature. Later on, the differences between the obtained strengths tended to decrease. Figure 8 shows the percentage difference in compressive strengths assuming that the strengths achieved by the heated specimens are 100%. After 7 days, the differences ranged from 78.5% to 66.1%. The results for the AP and PC specimens show greater differences. After 28 days, the differences in compressive strength decreased, falling within the 24.7% to 17.7% range. After this time, greater differences occurred in the compressive strength of the M and NF specimens. After 90 days, the difference in the strength of specimens cured at room temperature and those that were heated disappeared for the AP and PC specimens. For the M and NF specimens, they remained at the level of 27% to 30%.

At the same time, it is clear that the highest compressive strength results were obtained at each stage for the specimens in which the AP-based plasticiser was used. One of the reasons behind this result may be that they contained the smallest volume of air voids that weakened the matrix. Moreover, the strength of the APa and APe specimens reached similar values after 90 days. The same phenomenon can be observed in the case of the PCa and PCe specimens. It is also visible that the remaining specimens (M, N and PC) cured under the same conditions achieved similar strengths.

**Figure 9:**
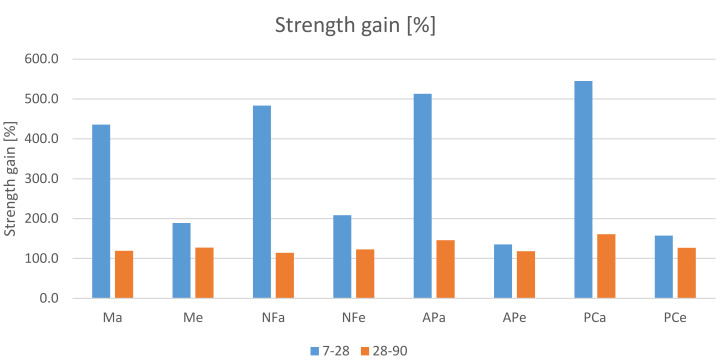
Percentage gain in compressive strength between the 7th and the 28th day and between the 28th and the 90th day.

The gain in strength between the 7th and the 28th day and between the 28th and the 90th day, shown in Figure 9, is also worth noting. It is clear that the gains observed for the specimens subjected to heating are more balanced. However, despite larger strength gains up to the 7th day for heated specimens and strength gains reaching 545% achieved by the unheated specimens between the 7th and 28th day, microscopic examination did not reveal any shrinkage cracks in any of the specimens.

**Figure 10:**
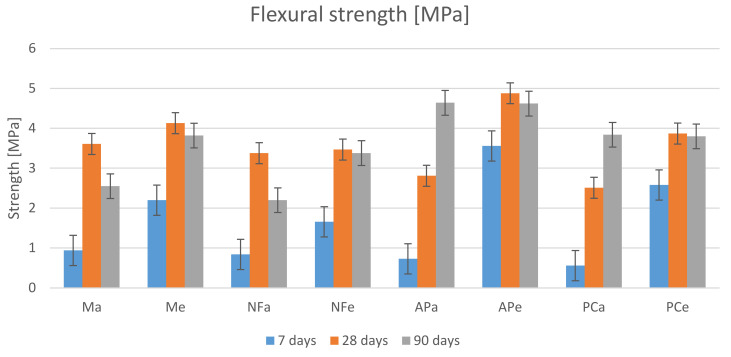
Flexural strength results.

Similarly to the results of the compressive strength tests, the highest value of flexural strength (Figure 10) was obtained for the mortar containing AP. The result for heated and unheated specimens after 90 days was identical—4.6 MPa. Additionally, the PC specimens achieved the same flexural strength values. In the case of the other additives, the results obtained for heated specimens were higher by 35%. The heated specimens tended to gain higher strength after 7 days as compared to those cured under laboratory conditions: Me—58%, NFe—50%, APe—80% and PCe—79%.

A phenomenon that did not occur in the case of compressive strength is the decrease in flexural strength of the specimens after 90 days. The reason behind this phenomenon requires further research. The loss of strength noted between the 28th and the 90th day for heated specimens is less than 8%; for Ma, it is 29.5% and for NFa, it is 35%. A stable gain in strength was only observed for the unheated APa and PCa specimens. Despite that, the results of the flexural strength test after 90 days ranged from 16.8% to 21.3% of the compressive strength test.

## 4. Conclusions

This study supplements the earlier results presented in the literature with regard to the outcome of research on the effect of plasticisers and their heating on the long-term strength results. Additionally, it investigates another acrylic-polymer-based additive which has not been considered in the literature thus far.

Among the plasticisers available on the domestic market, admixtures based on melamine, naphthalene and acrylic polymers had the greatest influence on the flow. The greatest penetration was observed for the M and NF specimens. The naphthalene-based additive had also the greatest impact on reducing the viscosity of the paste, but at the same time, it significantly increased the volume of air voids, which could result in strength loss. The highest compressive and flexural strengths were gained by specimens with an acrylic-polymer-based admix. Heating increased the early strength significantly; however, it had no effect on the compressive strength after 90 days for AP and PC additives. Nevertheless, the heating procedure increased the compressive strength of the M and NF specimens.

It is difficult to compare the research results obtained in this study with the results given in previous publications. This is due to imprecisely defined base components of the plasticisers. This, in turn, is due to the reluctance of manufacturers to share detailed information about their products. In most of the international literature, we can find polycarboxylate-based plasticisers. However, these can be either polycarboxylate compounds (acrylates) or polycarboxylic ethers. The same applies to the term acrylic, which can be given without specifying whether it refers to acrylates, acrylic acid copolymers or cross-linked acrylic polymers. All the published research results allow us to pick a plasticiser to achieve the expected results. The highest early and late strengths were obtained after curing samples acrylic-polymer-based superplasticiser in elevated temperatures. However, the increased curing temperature of the samples only influenced the early strength results. Its effect was not visible after 90 days. The AP addition also had a significant impact on improving the consistency of the mixture. However, further development in the area of geopolymer concrete additives requires additional studies that take into account the exact compositions of superplasticising agents currently available on the market.

## Figures and Tables

**Table 1:** Plasticisers used and the studies described in the referenced publications. Designations: PC—polycarboxylic ethers, M—melamine, NF—naphthalene, V—vinyl copolymers, L—lignosulfonates, + tested, − not tested, nd—no data.

Publication	Plasticiser					Slump	Viscosity	Curing Temperature		Strength				
	PC	M	NF	V	L	/Flow		Room	Elevated	1–3	7	14	28	90
Palacios, 2005	+	+	+	+	-	+	-	+	-	+	+	-	+	-
Criado, 2009	+	+	-	-	+	+	+	-	-	-	-	-	-	-
Nurrudin, 2011	+	-	-	-	-	+	-	-	+	+	+	-	+	-
Nurrudin, 2012	+	-	-	-	-	+	-	-	+	+	+	-	+	-
Montes, 2012	+	-	+	-	-	-	+	-	-	-	-	-	-	-
Laskar, 2013	+	-	-	-	+	+	+	-	-	-	-	-	-	-
Nematollahi, 2014	+	+	+	-	-	+	-	-	+	+	-	-	-	-
Jang, 2014	+	-	+	-	-	+	-	+	-	+	+	+	+	-
Xie, 2016	+	-	+	-	-	-	+	-	-	-	-	-	-	-
Salami, 2016	-	-	+	-	-	-	-	-	+	+	+	+	+	-
Jithendra, 2018	nd	nd	nd	nd	nd	+	-	+	-	+	-	-	+	-
Bong, 2019	+	-	+	-	-	+	-	-	+	+	-	-	-	-
Gupta, 2021	+	-	-	-	-	-	-	nd	nd	-	-	-	+	-

**Table 2:** Designations of the specimens and admixtures and specimen curing conditions.

Specimen Designation	Plasticiser Base	Curing Conditions
Ma	melamine (M)	20°
Me		70°
NFa	naphtalene (NF)	20°
NFe		70°
APa	acrylic polymers (AP)	20°
APe		70°
PCa	polycarboxylic ethers (PC)	20°
PCe		70°

## Data Availability

The data presented in this study are available on request from the corresponding author.
